# Association between the dietary literacy of children's daily diet providers and school-age children's nutritional status and eating behaviours: a cross-sectional study

**DOI:** 10.1186/s12889-022-14621-8

**Published:** 2022-12-06

**Authors:** Jun-Jie Chang, Nuo Xu, Ling-Ling Song, Yong-Han Li, Meng-Yuan Yuan, Ting-Ting Zhang, Yang He, Shan-Shan Chen, Geng-Fu Wang, Pu-Yu Su

**Affiliations:** 1 Department of Maternal, Child and Adolescent Health, School of Public Health, Anhui Medical University, No.81 Meishan Road, Anhui, 230032 China; 2 Maternal and Child Health Care and Family Planning Service Center Wujiang District of Suzhou City, No.551 Gaoxin Road, Jiangsu, 215200 China

**Keywords:** Eating behaviours, Nutritional status, School-age children, Dietary literacy

## Abstract

**Background:**

Overweight and obesity rates have increased rapidly in Chinese school-age children, and previous studies have indicated that poor dietary literacy can lead to unhealthy eating behaviours. However, few studies have investigated the association between the dietary literacy of daily diet providers and the eating behaviours and nutritional status of school-age children raised by the providers. Thus, we aimed to explore this association.

**Methods:**

We collected data on the eating behaviours and nutritional status of children in two primary schools in Anhui Province, as well as the dietary literacy of their daily diet providers. T-tests, one-way ANOVA, chi-square tests, and multiple linear regression were used to analyse the association.

**Results:**

We found significant differences in the scores on the Questionnaire of Children's Daily Diet Providers' Dietary Literacy (QCDDPDL) by region, relationship with the child, age, and educational level of the daily diet provider (all *p* < .05). Moreover, the children in the low QCDDPDL score group were inclined to engage in unhealthy eating behaviours such as emotional undereating and overeating (*p* < .05). In addition, the incidence of overweight and obesity was higher in the low QCDDPDL attitude score group than in the high score group (*p* = .006).

**Conclusions:**

Our study showed that the dietary literacy of diet providers may influence children's health and eating behaviours. Improving the dietary literacy of diet providers may promote the health status and eating behaviours of school-age children.

**Supplementary Information:**

The online version contains supplementary material available at 10.1186/s12889-022-14621-8.

## Introduction

The problem of overweight and obesity in children has become increasingly serious, affecting their current and even life-long health [1]. The 2014 Chinese National Survey of Student Health showed that the prevalence of overweight and obesity was 20.5% in students aged 7 to 18, which had increased approximately 3 times compared to two decades ago [2]. Obesity increases the risk of physical and psychological problems, including type 2 diabetes, cardiovascular and liver disease as well as social discrimination, low self-esteem, and depression [3–5]. Meanwhile, malnutrition cannot be neglected; although the prevalence of malnutrition in children and adolescents declined steadily over the last decade, it was up to 10% in economically underdeveloped areas of China in 2014 [6]. Malnutrition can not only stunt children but also increase susceptibility to infectious diseases [7]. Currently, the coexistence of malnutrition and obesity among children is a major public health problem in China [8]. A systematic review of obesity and dietary factors in children aged 6–12 years in the Middle East showed that obesity is related to missing breakfast, excessive intake of sugary beverages, and low consumption of fruits, vegetables and milk [9]. In addition, a cross-sectional study of school-age children aged 7–12 years in China found lower levels of magnesium, iron, and copper in the blood of children with picky eating behaviour [10]. Notably, extensive research has indicated that obesity in school-age children is associated with abnormal eating behaviours and specific appetite characteristics, which lead to the dysregulation of food varieties and intake [11, 12]. The Children’s Eating Behaviour Questionnaire (CEBQ) assesses a child's eating behaviours in 8 dimensions (satiety responsiveness, slowness in eating, food fussiness, food responsiveness, enjoyment of food, desire to drink, emotional undereating, and emotional overeating) based on parents' description of their child's daily eating activities [13]. The results of the systematic review and meta-analysis showed strong associations between overweight and obesity and eating behaviours such as overeating in children [14, 15]. Compared to normal-weight children, obese 11-year-old children showed faster eating, lower satiety, and other eating behaviours that may contribute to higher total food intake [16]. A study from the 'Growth and Obesity Chilean Cohort Study' (GOCS) in Chile also showed that emotional overeating is positively associated with children’s BMI in Chilean children aged 7–10 years [17]. Some studies have reported that the dietary literacy of parents is associated with children's eating behaviours and dietary quality [18, 19], suggesting that we could address children's obesity and malnutrition by strengthening the dietary literacy of their dietary providers [20].

Dietary literacy involves making healthy dietary decisions based on experience and knowledge about society, culture, food, and the environment [21]. The elements of dietary literacy include practical knowledge and skills to regulate food intake, such as selecting food and planning meals, and dietary literacy also includes the ability to understand the impact of food and nutrition on health [22]. A significant positive association between higher nutritional knowledge and higher intake of fruits and vegetables was found [23]. In addition, the majority of studies found positive associations between higher dietary literacy, such as more knowledge about food and more frequent food preparation behaviours, and healthier eating habits [24]. However, the lack of autonomy in food selection and long-term cohabitation make the dietary intake of school-age children largely dependent on the dietary provider. The attitudes, knowledge, and behaviours of diet providers will affect their food selections and meal preparation and in turn affect their children's eating behaviours and health status [25]. On the other hand, providers with low dietary literacy probably will not choose beneficial foods for their children by reading food labels and nutrient contents [26]. In addition, the family eating environment, for example, a reduction in screen time, could influence children's food intake [27]. Therefore, the dietary literacy of children's daily diet providers affects not only their health but also children's health and eating behaviours [28].

Most studies in the past have focused on the relationships between parental dietary literacy and infant feeding patterns, practices and nutritional status [29–32]. Importantly, not only does the nutritional status of the infant have a profound impact on future health [33, 34] but also the eating behaviours subsequently formed can lastingly impact the physical and mental health of children [35, 36]. However, few studies have examined the relationships between parents' dietary literacy and school-age children’s eating behaviours and nutritional status. At this age, eating behaviours and habits are being formed under the influence of their parents. The early establishment of healthy eating behaviours may promote children’s health and reduce the risk of various diseases later in life [37]. Particularly, there are a large number of migrant workers in China, and the care of their children may be left to other family members (http://www.stats.gov.cn/tjsj/zxfb/201902/t20190228_1651265.html). Therefore, we conducted this study to explore the association between the dietary literacy of daily diet providers and school-age children's nutritional status and eating behaviours in less economically developed areas of China.

## Methods

### Participants

One primary school from urban area and the other from rural area were selected for students who ate their daily meals at home. The schools are located in Hefei, Anhui Province, which is a less economically developed area in central China. The research was conducted in November 2018. All students in grades 3 to 6 were included in the investigation after informed consent was obtained from the children’s daily diet providers and assent was provided by the students. To obtain accurate information on the targeted population, we emphasized in the informed consent that the questionnaire should be completed by the person responsible for the child's diet, such as the one who routinely prepares the food. A total of 1,137 children and their daily diet providers participated in this study. The questionnaire was completed by the meal providers, and the children's physical development data and peripheral blood were collected. Those who did not complete the questionnaire and those who did not provide peripheral blood samples were excluded. The required max sample size was determined based on the chi-square test for medium effect size (w = 0.3), 5% significance level (two-tailed), 95% power, and a 4-DF (degree of freedom), and the compute required sample size was 207 [38]. Ethics approval was given by the Biomedicine Ethics Committee of Anhui Medical University (No. 20170386).

## Questionnaire

A self-report questionnaire was completed by the student's daily diet provider. The questionnaire mainly includes three parts: 1. General demographic characteristics of the student and her/his dietary provider, such as the child's age, life area (urban, rural), sex (male, female), and the dietary provider’s age, relationship with child, education level and self-rated family economy. 2. The dietary literacy of children's daily diet providers was evaluated by the Questionnaire of Children's Daily Diet Providers' Dietary Literacy (QCDDPDL), which was developed by our research group and was verified with acceptable reliability and validity [39]. The QCDDPDL consists of 30 items that investigate the dietary literacy of daily diet providers from four dimensions, namely, attitude (7 items, e.g., Pay attention to choose fresh and hygienic food), actions (7 items, e.g., Make sure your children drink milk every day), skills (9 items, e.g., Can understand the nutrition label on food packaging), and environment (7 items, e.g., Can keep home dining table and tableware clean), by using a 4-point Likert scale (0 = very inconsistent, 3 = very consistent). Five of these items are reverse scored (Additional file 1: Supplement Table 1). In this study, the score of the four dimensions was calculated by adding their corresponding items, and the total score was calculated by summing the scores of the four dimensions. A higher score indicates a stronger ability of the child’s daily diet provider to use dietary nutrition knowledge to improve the child's health status. The Cronbach’s α coefficient, split-half reliability, and test–retest reliability of the QCDDPDL were 0.874, 0.813, and 0.878, respectively. Additionally, the structural equation model constructed using AMOS demonstrated that the QCDDPDL had an acceptable validity: goodness-of-fit index (GFI = 0.927), adjusted goodness-of-fit index (AGFI = 0.912), and root mean square error of approximation (RMSEA = 0.042). 3. The children’s eating behaviours were assessed by using the Children's Eating Behaviour Questionnaire (CEBQ), which was developed by the British scholar Wardle and colleagues in 2001 and validated as having acceptable reliability and validity [13]. The CEBQ contains 35 items that evaluate children's eating behaviours from 8 dimensions, namely, satiety responsiveness (5 items, e.g., My child gets full up easily), slowness in eating (4 items, e.g., My child eats slowly), food fussiness (6 items, e.g., My child enjoys a wide variety of foods), food responsiveness (5 items, e.g., My child’s always asking for food), enjoyment of food (4 items, e.g., My child enjoys eating), desire to drink (3 items, e.g., My child is always asking for a drink), emotional undereating (4 items, e.g., My child eats less when s/he is upset), and emotional overeating (4 items, e.g., My child eats more when anxious). The CEBQ items are scored on a 5-point Likert scale (1 = never, 5 = always), and the score of each dimension is calculated by summing its corresponding items. A higher score indicates stronger characteristics of the corresponding dimension. In this study, 64 school-age children (2 students per classroom) were retested 1 week after the survey, and the retest reliability of the questionnaire score ranged from 0.86 to 0.93 for each dimension.

## Detection of children's nutritional status

### BMI and body fat percentage

The weight and height of children were measured to calculate BMI using the formula BMI = weight (kg)/height^2^ (m^2^). Students were divided into 3 categories (wasting, normal, and overweight or obese) by using the BMI cut-off recommended by the World Health Organization [40]. For a detailed division, see Additional file 2: Supplement Table 2. In addition, the skinfold thickness of the upper triceps and subscapular folds was measured by sebaceous callipers. The measured skinfold thickness was converted into body fat density using the Japanese Changling formula, and then the percentage of body fat was estimated using the modified formula of Brozek [41]. The body fat percentage was divided by quartile. *P*_25_ to *P*_75_ was considered the normal body fat group, < *P*_25_ was considered the low body fat group, and > *P*_75_ was considered the high body fat group [42].

### Haemoglobin

A haemoglobin strip (dry chemical method) was used to test the haemoglobin content in the blood from the fingertips of children. Hb < 115 g/L in children aged 5 to 11 and Hb < 120 g/L in children aged 12 to 14 were used as the defining criteria for nutritional anaemia in children based on the WHO recommended standards [43].

### Calcium, Iron, and Zinc

Forty microliters of blood from the fingertips of children were aspirated by using a micropipette and measured within 2 h after shaking. The contents of calcium (Ca), iron (Fe), and zinc (Zn) elements in the sample were detected by the standard curve method using a BH5500 atomic absorption spectrometer (Beijing Bohui Innovative Photoelectric Technology Co., Ltd., Beijing). The children’s element contents were divided into normal and deficient groups by the detection criteria. The reference ranges of normal values of Ca, Fe, and Zn in children aged 6 to 11 years were 1.51 to 2.01 mmol/L, 7.36 to 9.34 mmol/L, and 67.72 to 103.84 µmol/L, respectively; the reference ranges of normal values of Ca, Fe, and Zn in children aged 12 to 14 years were 1.42 to 1.90 mmol/L, 7.16 to 9.85 mmol/L, and 71.46 to 111.33 µmol/L, respectively. Data of reference ranges were provided by the measurement company using a BH5500 atomic absorption spectrometer [44].

### Quality control

The children took the questionnaire home after school (an instruction manual was attached to guide the diet provider); the questionnaire was completed by their guardians, who mainly provided meals for them and were returned the next morning. The head teachers were in charge of this process. The physical development of students was measured uniformly in schools by child health workers from our university-affiliated hospital after obtaining informed consent. Biological indicators of blood samples from the two schools were tested in one laboratory, all instruments for laboratory testing were calibrated, and the testing was completed by laboratory professionals.

### Statistical analysis

The database was established by using EpiData 3.1, and SPSS 21.0 was used for statistical analysis. First, t-test and one-way ANOVA were used to compare the dietary literacy scores of daily diet providers based on different demographic characteristics. Then, the dietary literacy score of each dimension and the total score were divided into the low group (< *P*_25_), moderate group (*P*_25_ to *P*_75_), and high group (> *P*_75_) by using the quartile method [45]. Chi-square tests were used to explore whether there were differences in the nutritional status of children among different dietary literacy groups. Third, one-way ANOVA was used to compare whether there were differences in 8 dimensions of children’s eating behaviours between the three dietary literacy groups. Last, multiple linear regression analysis (controlling for demographic characteristics) was used to explore the effects of daily diet providers' dietary literacy on school-age children's eating behaviours. *p* < 0.05 was considered significant.

## Results

Ultimately, 1,043 children and their main diet providers were included because of the complete questionnaire and complete data on children’s nutritional status. The effective rate was 91.7%, and there were no significant differences in sex or BMI between the 1,043 included children and the 94 non-included children (*p* > 0.05). In addition, no significant differences were found between the included diet providers and the non-included diet providers in region, relationship with child, age, education level, and self-rated family economy level (*p* > 0.05). Among the included children, 693 (66.4%) were from urban areas, and 350 (33.6%) were from rural areas; boys and girls accounted for almost half each, and their ages ranged from 7 to 13 years, with a mean of 9.89 ± 1.32 years. Table 1 shows that there were significant differences in the four dimensions and total score between the daily diet provider’s region, relationship with the child, age, and educational level. However, there were no significant differences in the attitude and environment dimensions between the self-rated family economy levels. The dietary literacy scores of the medium upper self-rated family economy were significantly higher than those of the lower family economy only in behaviour, skill, and total score.

**Table 1:** Comparison of daily diet providers’ dietary literacy scores between different demographic characteristics

Characteristics	N (%)	Attitude Mean SD	Behaviour Mean SD	Skill Mean SD	Environment Mean SD	Total score Mean SD
**Region**						
Cities	693(66.4)	15.0 2.7	15.3 3.5	19.0 4.1	14.0 2.8	63.2 10.3
Countryside	350(33.6)	13.6 3.2	14.3 3.9	18.1 4.5	12.8 3.2	58.7 11.5
* p*		< .001	< .001	.001	< .001	< .001
**Relationship with child**						
Parents	894(85.7)	14.7 2.8	15.1 3.5	18.9 4.1	13.8 2.8	62.5 10.4
Grandparents	128(12.3)	13.2 3.4	13.7 4.1	17.3 4.9	12.4 3.5	56.6 12.7
Other	21(2.0)	13.1 3.6	13.8 4.5	19.0 4.6	12.6 3.7	58.3 13.3
* p*		< .001	< .001	< .001	< .001	< .001
**Diet provider age**						
< 30 years	44(4.2)	14.4 3.1	14.6 4.1	19.0 4.7	13.1 3.3	61.0 12.6
30 ~ 40 years	649(62.2)	14.8 2.8	15.2 3.4	19.0 4.0	13.9 2.8	62.9 10.1
40 ~ 50 years	223(21.4)	14.3 3.0	15.0 3.7	18.4 4.4	13.4 3.1	61.1 11.2
50 ~ 60 years	47(4.5)	13.5 3.5	14.5 4.6	18.3 4.3	13.3 3.5	59.5 12.5
> 60 years	80(7.7)	13.2 3.2	13.3 3.8	16.9 4.9	12.0 3.3	55.3 12.1
* p*		< .001	< .001	< .001	< .001	< .001
**Education**						
Primary school or lower	197(18.9)	13.3 3.2	13.8 4.0	17.8 4.8	12.4 3.1	57.2 12.0
Junior middle school	417(40.0)	14.6 3.0	15.0 3.5	18.7 4.2	13.9 2.8	62.2 10.4
High school	233(22.3)	15.0 2.6	15.5 3.8	19.3 4.1	13.9 3.0	63.6 11.1
College and higher	196(18.8)	14.9 2.6	15.3 3.1	18.9 3.7	13.8 2.8	62.9 9.5
* p*		< .001	< .001	.001	< .001	< .001
**Self-rated family economy**						
Medium lower	236(22.6)	14.3 3.0	14.2 3.8	18.1 4.7	13.2 3.1	59.8 11.6
Medium	755(72.4)	14.5 3.0	15.0 3.5	18.8 4.1	13.7 2.9	62.1 10.6
Medium upper	52(5.0)	15.0 2.9	16.2 3.9	19.6 4.4	14.0 3.0	64.7 11.5
* p*		.277	< .001	< .001	.063	.002

Table 2 shows that there was no significant difference in the distribution of the percentage of body fat or haemoglobin content among the different dietary literacy groups. Only in the dimension of attitude was a significant difference found between the distribution of children’s BMI and the dietary literacy of daily diet providers (*p* = 0.006); in the remaining dimensions, no significant differences were found. For comparison of Ca, Fe, and Zn among different dietary literacy groups, Table 3 shows that the contents of calcium, iron, and zinc did not differ significantly among children in the low, moderate, and high dietary literacy groups for each dimension and the total score. Table 4 shows that children raised by diet providers with high total dietary literacy had lower scores in various eating behaviours except for slowness in eating and enjoyment of food compared with those raised by diet providers with low total dietary literacy. The scores for slowness in eating were also lower, which was not statistically significant. Notably, a higher score for children’s enjoyment of food was found in the high total dietary literacy group.

**Table 2:** Comparison of partial nutritional status of children in different dietary literacy groups

Dietary literacy	N (%)	Body fat percentage^a^					BMI^b^			Haemoglobin^c^		
		**Normal**		**High**		**Low**	**Normal**	**Wasting**	**Overweight** **or obesity**	**Normal**		**Deficiency**
**Attitude**												
Low score < 13	233(22.3)	109		63		61	147	21	65	219		14(6.0)
Moderate score 13–17	652(62.5)	327		166		159	483	31	138	590		62(9.5)
High score > 17	158(15.2)	79		35		44	103	14	41	142		16(10.1)
* χ*^2^		2.00					14.29			3.01		
* p*		.735					.006			.222		
**Behaviour**												
Low score < 13	246(23.6)	116		66		64	178	12	56	226		20(8.1)
Moderate score 13–18	610(58.5)	306		153		151	432	39	139	554		56(9.2)
High score > 18	187(17.9)	93		45		49	123	15	49	171		16(8.6)
* χ*^2^		0.86					3.15			0.26		
* p*		.930					.532			.878		
**Skill**												
Low score < 16	222(21.3)	109		58		55	157	17	48	202		20(9.0)
Moderate score 16–22	605(58.0)	290		162		153	420	40	145	551		54(8.9)
High score > 22	216(20.7)	116		44		56	156	9	51	198		18(8.3)
* χ*^2^		3.79					2.87			0.08		
* p*		.435					.581			.960		
**Environment**												
Low score < 12	251(24.1)	121		65		65	183	16	52	228		23(9.2)
Moderate score 12–16	614(58.9)	294		160		160	429	39	146	557		57(9.3)
High score > 16	178(17.0)	100		39		39	121	11	46	166		12(6.7)
* χ*^2^		3.98					1.67			1.16		
* p*		.409					.797			.561		
**Total score**												
Low score < 55	258(24.7)	125		66		67	183	16	59	236		22(8.5)
Moderate score 55–70	550(52.7)	261		153		136	393	39	118	500		50(9.1)
High score > 70	235(22.6)	129		45		61	157	11	67	215		20(8.5)
* χ*^2^		6.96					5.59			0.11		
* p*		.138					.232			.949		

^**a**^*P*_25_-*P*_75_ was used as normal group, < *P*_25_ as low body fat group, > *P*_75_ as high body fat group; ^***b***^Detailed BMI classification was shown in Supplemental Table 2; ^***c***^HB < 115 g/L in children aged 5 to 11 and Hb < 120 g/L in children older than 11 were considered as deficiency

**Table 3:** Comparison of trace elements status among children in different dietary literacy groups

Dietary literacy	N (%)	Calcium		Iron		Zinc		
		**Normal**	**Deficiency**	**Normal**	**Deficiency**	**Normal**	**Deficiency**	
**Attitude**								
Low score	233(22.3)	192	41	189	44	213	20	
Moderate score	652(62.5)	494	158	533	119	565	87	
High score	158(15.2)	126	32	131	27	141	17	
χ^2^		4.77		0.20		3.94		
* p*		.092		.903		.140		
**Behaviour**								
Low score	246(23.6)	191	55	203	43	213	33	
Moderate score	610(58.5)	480	130	497	113	537	73	
High score	187(17.9)	141	46	153	34	169	18	
χ^2^		0.91		0.13		1.47		
* p*		.636		.938		.481		
**Skill**								
Low score	222(21.3)	178	44	177	45	185	37	
Moderate score	605(58.0)	469	136	498	107	541	64	
High score	216(20.7)	165	51	178	38	193	23	
χ^2^		1.01		0.80		6.15		
* p*		.605		.671		.146		
**Environment**								
Low score	251(24.0)	198	53	211	40	224	27	
Moderate score	614(58.9)	474	140	500	114	540	74	
High score	178(17.1)	140	38	142	36	155	23	
χ^2^		0.37		1.41		0.50		
* p*		.830		.495		.777		
**Total score**								
Low score	258(24.7)	206	52	212	46	228	30	
Moderate score	550(52.7)	429	121	451	99	479	71	
High score	235(22.6)	177	58	190	45	212	23	
χ^2^		1.48		0.18		1.55		
* p*		.478		.914		.460		

The cut-offs of dietary literacy are the same as Table 2; Reference ranges of normal values of Ca, Fe, and Zn in children aged 6 to 11 years were 1.51 to 2.01 mmol/L, 7.36 to 9.34 mmol/L, and 67.72 to 103.84 µmol/L, respectively; Reference ranges of normal values of Ca, Fe, and Zn in children aged 12 to 14 years were 1.42 to 1.90 mmol/L, 7.16 to 9.85 mmol/L, and 71.46 to 111.33 µmol/L, respectively

**Table 4:** Comparison of eating behaviours of children in different dietary literacy of daily diet providers

Dietary literacy	Satiety responsiveness Mean SD	Slowness in eating Mean SD	Food fussiness Mean SD	Food responsiveness Mean SD	Enjoyment of food Mean SD	Desire to drink Mean SD	Emotional undereating Mean SD	Emotional overeating Mean SD
**Attitude**								
Low score	12.7 3.7	10.3 3.5	16.1 4.1	11.4 4.2	13.1 3.8	6.6 3.0	10.3 3.3	8.2 3.5
Moderate score	12.4 3.3	10.0 3.2	15.2 4.1	10.4 3.9	13.8 3.7	6.1 2.9	10.0 3.1	7.2 3.0
High score	11.9 2.9	9.6 3.2	14.2 4.2	10.1 4.0	14.5 4.1	5.9 2.8	9.8 3.4	6.3 2.9
* F*	3.17	1.79	9.74	6.77	6.64	2.91	1.06	18.6
* p*	.042	.167	< .001	.001	.001	.055	.347	< .001
**Behaviour**								
Low score	12.8 3.5	10.1 3.3	15.9 4.1	11.1 4.0	13.2 3.7	6.7 3.1	10.3 3.3	7.9 3.2
Moderate score	12.4 3.2	10.1 3.3	15.3 4.0	10.6 4.0	13.8 3.6	6.1 2.8	10.0 3.1	7.2 3.1
High score	11.9 3.5	9.8 3.3	14.3 4.6	9.7 3.6	14.2 4.3	5.8 3.0	9.7 3.4	6.7 3.1
* F*	3.85	0.77	7.82	7.11	3.80	5.74	1.84	7.84
* p*	.022	.466	< .001	.001	.023	.003	.159	< .001
**Skill**								
Low score	12.9 3.3	10.5 3.5	16.7 4.3	11.1 4.0	13.4 3.5	7.0 3.2	10.1 3.4	7.5 3.4
Moderate score	12.6 3.3	10.0 3.3	15.2 3.8	10.7 3.9	13.6 3.6	6.1 2.7	10.1 3.1	7.3 3.1
High score	12.0 3.4	9.7 3.0	14.0 4.4	9.7 4.0	14.5 4.3	5.6 3.0	9.7 3.4	7.0 3.2
* F*	3.74	4.23	25.47	7.28	5.56	12.72	1.26	1.94
* p*	.024	.015	< .001	.001	.004	< .001	.283	.144
**Environment**								
Low score	13.0 3.6	10.3 3.3	16.0 4.0	11.2 4.2	13.0 3.8	6.5 2.9	10.2 3.2	7.9 3.4
Moderate score	12.4 3.3	10.0 3.4	15.2 4.2	10.6 3.9	13.9 3.7	6.3 3.0	10.1 3.2	7.2 3.0
High score	11.6 3.0	9.9 3.0	14.6 4.2	9.5 3.8	14.1 4.0	5.3 2.6	9.3 3.3	6.6 3.2
* F*	8.81	0.87	5.95	10.22	6.92	10.90	5.88	9.44
* p*	< .001	.419	.003	< .001	.001	< .001	.003	< .001
**Total score**								
Low score	12.9 3.6	10.3 3.3	16.2 4.1	11.6 4.0	13.2 3.5	6.6 3.0	10.4 3.4	8.1 3.4
Moderate score	12.4 3.3	10.0 3.3	15.3 4.0	10.6 4.0	13.7 3.7	6.2 2.8	10.0 3.8	7.2 3.0
High score	11.8 3.1	9.8 3.2	14.2 4.5	9.4 3.6	14.4 4.2	5.6 3.0	9.6 3.4	6.6 3.0
* F*	6.96	1.30	14.05	18.73	6.12	6.99	3.35	14.85
* p*	.001	.274	< .001	< .001	.002	.001	.036	< .001

The cut-offs of dietary literacy are the same as Table 2; The df of analysis of variance between groups and within groups are 2 and 1040, respectively

Table 5 shows that the high total score group of providers’ dietary literacy was associated with reduced scores of most eating behaviours (satiety responsiveness, slowness in eating, food fussiness, food responsiveness, desire to drink, emotional undereating, and emotional overeating) (*β* < 0, *p* < 0.05). However, the high total score group was associated with increased scores of children’s enjoyment of food (*β* > 0, *p* < 0.05). Even if the effects on eating behaviours were not as strong as the high score group, the moderate score group also showed significant associations with food fussiness, food responsiveness, desire to drink, and emotional overeating.

**Table 5:** Effect of daily diet providers' dietary literacy score on children's eating behaviours

Dietary literacy	Satiety responsiveness		Slowness in eating		Food fussiness		Food responsiveness		Enjoyment of food		Desire to drink		Emotional undereating		Emotional overeating	
	***β***	***p***	***β***	***p***	***β***	***p***	***β***	***p***	***β***	***p***	***β***	***p***	***β***	***p***	***β***	***p***
**Attitude**																
Moderate score	-0.02	.535	-0.06	.126	-0.09	**.023**	-0.10	**.010**	0.08	**.040**	-0.09	**.015**	-0.04	.342	-0.13	** < .001**
High score	-0.07	.053	-0.10	**.010**	-0.15	** < .001**	-0.09	**.011**	0.12	**.002**	-0.10	**.005**	-0.04	.271	-0.20	** < .001**
**Behaviour**																
Moderate score	-0.04	.334	-0.01	.958	-0.06	.109	-0.04	.310	0.07	.074	-0.12	**.001**	-0.05	.194	-0.09	**.014**
High score	-0.08	**.036**	-0.05	.223	-0.14	** < .001**	-0.12	**.002**	0.08	**.035**	-0.14	** < .001**	-0.07	.080	-0.12	**.001**
**Skill**																
Moderate score	-0.07	.082	-0.10	**.007**	-0.19	** < .001**	-0.03	.478	0.02	.561	-0.16	** < .001**	-0.01	.907	-0.02	.671
High score	-0.09	**.023**	-0.12	**.003**	-0.27	** < .001**	-0.13	**.001**	0.10	**.008**	-0.20	** < .001**	-0.05	.164	-0.07	.094
**Environment**																
Moderate score	-0.07	.074	-0.03	.375	-0.10	**.008**	-0.07	.064	0.11	**.004**	-0.04	.249	-0.01	.771	-0.10	**.006**
High score	-0.13	** < .001**	-0.06	.095	-0.12	**.002**	-0.15	** < .001**	0.09	**.011**	-0.18	** < .001**	-0.11	**.005**	-0.14	** < .001**
**Total score**																
Moderate score	-0.06	.116	-0.05	.175	-0.11	**.004**	-0.10	**.006**	0.05	.157	-0.09	**.014**	-0.05	.167	-0.12	**.002**
High score	-0.11	**.005**	-0.08	**.031**	-0.19	** < .001**	-0.20	** < .001**	0.11	**.006**	-0.17	** < .001**	-0.09	**.015**	-0.18	** < .001**

The groups of dietary literacy are the same as Table 2 and the low score group was taken as the reference group. Region, relationship with child, diet provider age, education, and self-rated family were controlled as covariates

## Discussion

The first finding of this study was that children’s diet providers who were in the city, younger, highly educated, and economically advanced had a higher level of dietary literacy. Such phenomena are easily understandable. For urban children who live in better-educated families, their daily diet providers pay more attention to dietary nutrition and purchase more suitable food for children's growth and development [46]. Those with better economic status can choose a wider variety of food with better quality. Well-educated people can make safer, healthier food choices and are consciously maintaining balance diets that are beneficial to their bodies, such as consuming more vegetables and fruits; also, they will encourage their children to eat more vegetables and fruits [47–49]. Therefore, the dietary literacy scores of these people were higher on attitude, action, skills, and environment dimensions compared to lower educational and economic level. However, no correlation was found between the nutritional status of the child (body fat percentage, haemoglobin, calcium, iron, zinc) and the dietary literacy of their diet providers in these two primary schools. People could purchase most of the food they needed for the rapid socioeconomic development of China in the last two decades, which was the main reason contributing to improving children’s nutritional status. The effects of economic development are particularly strong in economically disadvantaged rural areas [2]. People with high dietary literacy are more likely to provide dietary supplements to their children; theoretically, children who consume dietary supplements will have higher levels of micronutrients than those who do not [50]. However, no differences were found in Ca, Fe, and Zn contents between different dietary literacy groups in our study. A Korean study also did not find significant differences in most dietary macronutrients and micronutrients between dietary supplement (DS) users and nonusers [51]. One possible reason is that children in the high dietary literacy group also consume more vegetables and fruits, which hinders the absorption of micronutrients such as calcium [52]. Notably, although there were no significant differences between the daily dietary literacy score of providers and children's BMI in most dimensions, it was statistically significant in the attitude dimension. A reasonable explanation was that the high dietary literacy score of providers who find their children overweight would take measures to prevent their children from being overweight and obese [53].

The scores of the dietary literacy attitude, behaviour, skill, and environment dimensions and the total dietary literacy score of school-age children's daily diet providers were significantly correlated with most eating behaviours of the children. Apart from enjoyment of food, the remaining seven eating behaviours all scored lower in the high dietary literacy group than in the low dietary literacy group. Furthermore, the multiple linear regression results suggested that the total score of daily dietary literacy of diet providers was negatively associated with children's satiety responsiveness, slowness in eating, food fussiness, food responsiveness, desire to drink, emotional undereating, and emotional overeating behaviours but positively associated with children's enjoyment of food. More than one study showed that emotional overeating or undereating in early childhood is not genetic [54, 55]; rather, it is influenced by the family environment. If a pleasant environment is not ensured at meal times, the child will develop the eating behaviour of undereating [56]. This is consistent with the findings of our study that the environmental dimension of dietary providers was negatively associated with children’s emotional undereating. Similarly, studies on picky eating in young children also suggested that parents repeatedly offering unfamiliar and unwanted foods to their children in a stressful situation could lead to food fussiness [57, 58]. However, in our study, the dietary literacy of the diet provider was positively associated with their child's enjoyment of food, which may be related to the ability of high dietary literacy providers to make tasty food. It is worth noting that higher food enjoyment is associated with higher risks of being obese [59]. Based on previous research and our study, dietary literacy of diet providers is likely to be associated with the eating behaviours of school-age children. In addition, our study showed that the effects of the high dietary literacy group were stronger than those of the moderate dietary literacy group, suggesting that the higher the dietary literacy of the dietary provider was, the greater the effects on the child's eating behaviours. In the current study, compared to nutritional status, the associations between school-age children’s eating behaviours and the diet provider’s dietary literacy were more apparent. Previous studies showed that obese children were more sensitive to the smell of food and more likely to overeat after exposure to preferred foods than normal-weight children [60]. Overreaction to food may not be limited to food choices but may also include beverages, especially sugar-sweetened beverages, which are associated with weight gain [61]. The results of a meta-analysis showed that children’s eating behaviours, such as food responsiveness, food enjoyment, emotional overeating, and desire to drink, could lead to obesity [11]. Obese children may remain obese into adulthood and be more likely to develop noncommunicable diseases such as diabetes and cardiovascular disease early in life [62]. Therefore, we can intervene in children's eating behaviours to prevent childhood obesity by improving the dietary literacy of diet providers. First, making diet providers aware of the importance of a healthy diet for children's physical development, namely, improving the attitude dimension. Second, suggesting diet providers provide children with a balanced and nutritious meal every day, namely, improving the action dimension. Third, cultivating diet providers' knowledge of nutrition so that they can know the nutritional content of various foods, namely, improving the skills dimension. Fourth, reminding diet providers of the need for a pleasant environment when eating, namely, improving the environment dimension. Some nutrition education programmes for parents have shown promising results with a marked improvement in the dietary literacy of trained people [63–65]. Future research could explore the relationships between dietary literacy of diet providers and children's eating behaviours based on establishing cohorts, and could also explore the impacts of improving diet providers’ dietary literacy on children's eating behaviours.

### Limitations

There were some deficiencies in this study. First, more than one person may provide daily diets for their child, such as when both parents participate; however, this study only investigated the dietary literacy of one provider, and the results may not be sufficiently accurate. Second, measurement bias and social desirability bias may exist, the CEBQ is a subjective scale based on parental reports, and our study only captured the opinion of one diet provider, reports of children's eating behaviours may be biased, furthermore, diet providers may mask their child's poor eating habits to conform to social expectations, future studies could attempt to measure children's eating behaviours in objective ways such as applying cameras. Third, cross-sectional studies limit causality, and further cohort studies or randomized controlled studies can be conducted to examine the relationships between diet providers’ dietary literacy and children's eating behaviours. In addition, due to dietary culture, religious beliefs, and other factors, there are large differences in food choices in different regions. This study only conducted a sample survey in one region, and the survey results cannot be generalized. It is necessary to survey a larger sample population in future studies.

## Conclusions

In this study, the dietary literacy of school-age children's daily diet providers was associated with children’s eating behaviours. Although no association was found between the nutritional status of children and the dietary literacy of their dietary providers, previous studies have shown that unhealthy eating behaviours increase the risk of some chronic diseases. Therefore, we can improve the dietary literacy of children's diet providers to cultivate children's healthy eating behaviours. Additionally, appropriate intervention measures, such as improving diet providers’ dietary literacy through education programs, should be formulated with full consideration of socioeconomic and educational levels to reduce the risk of children's nutrition-related diseases and promote children's physical and mental health. In addition, based on the proposed interventions, community trials can be carried out to evaluate the effects of the interventions.

## Supplementary Information

**Additional file 1.**

**Additional file 2.**

## Data Availability

The data presented in this study are available upon request to the corresponding author.

## Abbreviations

QCDDPD: Questionnaire of Children's Daily Diet Providers' Dietary Literacy
CEBQ: Children's Eating Behaviour Questionnaire
BMI: Body mass index
ANOVA: Analysis of Variance

## References

[1] Valerio G, Licenziati MR, Manco M, Ambruzzi AM, Bacchini D, Baraldi E (2014). Health consequences of obesity in children and adolescents. Minerva Pediatr.

[2] Dong Y, Jan C, Ma Y, Dong B, Zou Z, Yang Y (2019). Economic development and the nutritional status of Chinese school-aged children and adolescents from 1995 to 2014: an analysis of five successive national surveys. Lancet Diabetes Endocrinol.

[3] Maggio CA, Pi-Sunyer FX (2003). Obesity and type 2 diabetes. Endocrinol Metab Clin North Am.

[4] Fields LC, Brown C, Skelton JA, Cain KS, Cohen GM (2021). Internalized Weight Bias, Teasing, and Self-Esteem in Children with Overweight or Obesity. Childhood obesity (Print).

[5] Rao WW, Zong QQ, Zhang JW, An FR, Jackson T, Ungvari GS (2020). Obesity increases the risk of depression in children and adolescents: Results from a systematic review and meta-analysis. J Affect Disord.

[6] Dong YH, Wang ZH, Yang ZG, Wang XJ, Chen YJ, Zou ZY, et al. Epidemic status and secular trends of malnutrition among children and adolescents aged 7–18 years from 2005 to 2014 in China. Journal of Peking University Health sciences. 2017; 49(3):424–32. https://kns.cnki.net/kcms/detail/detail.aspx?FileName=BYDB201703009&DbName=CJFQ2017 28628142

[7] Owino VO, Murphy-Alford AJ, Kerac M, Bahwere P, Friis H, Berkley JA (2019). Measuring growth and medium- and longer-term outcomes in malnourished children. Matern Child Nutr.

[8] Zhou S, Ye B, Fu P, Li S, Yuan P, Yang L (2020). Double Burden of Malnutrition: Examining the Growth Profile and Coexistence of Undernutrition, Overweight, and Obesity among School-Aged Children and Adolescents in Urban and Rural Counties in Henan Province. China Journal of obesity.

[9] Albataineh SR, Badran EF, Tayyem RF (2019). Dietary factors and their association with childhood obesity in the Middle East: A systematic review. Nutr Health.

[10] Xue Y, Lee E, Ning K, Zheng Y, Ma D, Gao H (2015). Prevalence of picky eating behaviour in Chinese school-age children and associations with anthropometric parameters and intelligence quotient. A cross-sectional study Appetite.

[11] Kininmonth A, Smith A, Carnell S, Steinsbekk S, Fildes A, Llewellyn C (2021). The association between childhood adiposity and appetite assessed using the Child Eating Behavior Questionnaire and Baby Eating Behavior Questionnaire: A systematic review and meta-analysis. Obesity reviews: an official journal of the International Association for the Study of Obesity.

[12] Sun M, Hu X, Li F, Deng J, Shi J, Lin Q (2020). Eating Habits and Their Association with Weight Status in Chinese School-Age Children: A Cross-Sectional Study. Int J Environ Res Public Health.

[13] Wardle J, Guthrie CA, Sanderson S, Rapoport L (2001). Development of the Children's Eating Behaviour Questionnaire. J Child Psychol Psychiatry.

[14] He J, Cai Z, Fan X (2017). Prevalence of binge and loss of control eating among children and adolescents with overweight and obesity: An exploratory meta-analysis. Int J Eat Disord.

[15] Kininmonth A, Smith A, Carnell S, Steinsbekk S, Fildes A, Llewellyn C (2021). The association between childhood adiposity and appetite assessed using the Child Eating Behavior Questionnaire and Baby Eating Behavior Questionnaire: A systematic review and meta-analysis. Obes Rev.

[16] Barkeling B, Ekman S, Rössner S (1992). Eating behaviour in obese and normal weight 11-year-old children. International journal of obesity and related metabolic disorders: journal of the International Association for the Study of Obesity.

[17] Sánchez U, Weisstaub G, Santos JL, Corvalán C, Uauy R (2016). GOCS cohort: children's eating behavior scores and BMI. Eur J Clin Nutr.

[18] Nakamura T, Akamatsu R, Yoshiike N (2021). Mindful Eating Proficiency and Healthy Eating Literacy among Japanese Mothers: Associations with Their Own and Their Children's Eating Behavior. Nutrients.

[19] Gibbs HD, Kennett AR, Kerling EH, Yu Q, Gajewski B, Ptomey LT (2016). Assessing the Nutrition Literacy of Parents and Its Relationship With Child Diet Quality. J Nutr Educ Behav.

[20] Feng A, Wang L, Chen X, Liu X, Li L, Wang B (2015). Developmental Origins of Health and Disease (DOHaD): Implications for health and nutritional issues among rural children in China. Biosci Trends.

[21] Vidgen HA, Gallegos D (2014). Defining food literacy and its components. Appetite.

[22] Krause C, Sommerhalder K, Beer-Borst S, Abel T (2018). Just a subtle difference? Findings from a systematic review on definitions of nutrition literacy and food literacy. Health Promot Int.

[23] Spronk I, Kullen C, Burdon C, O'Connor H (2014). Relationship between nutrition knowledge and dietary intake. Br J Nutr.

[24] Vaitkeviciute R, Ball LE, Harris N (2015). The relationship between food literacy and dietary intake in adolescents: a systematic review. Public Health Nutr.

[25] Ek A, Sorjonen K, Eli K, Lindberg L, Nyman J, Marcus C (2016). Associations between Parental Concerns about Preschoolers' Weight and Eating and Parental Feeding Practices: Results from Analyses of the Child Eating Behavior Questionnaire, the Child Feeding Questionnaire, and the Lifestyle Behavior Checklist. PLoS ONE.

[26] Ma J, Zhu Z, Chen X, Guo Y, Zhang H, Zhang Y (2018). A cross-sectional survey of nutrition labelling use and its associated factors on parents of school students in Shanghai. China Public health nutrition.

[27] Fulkerson JA, Friend S, Horning M, Flattum C, Draxten M, Neumark-Sztainer D (2018). Family Home Food Environment and Nutrition-Related Parent and Child Personal and Behavioral Outcomes of the Healthy Home Offerings via the Mealtime Environment (HOME) Plus Program: A Randomized Controlled Trial. J Acad Nutr Diet.

[28] Fadare O, Amare M, Mavrotas G, Akerele D, Ogunniyi A (2019). Mother's nutrition-related knowledge and child nutrition outcomes: Empirical evidence from Nigeria. PLoS ONE.

[29] Yin HS, Sanders LM, Rothman RL, Shustak R, Eden SK, Shintani A (2014). Parent health literacy and "obesogenic" feeding and physical activity-related infant care behaviors. J Pediatr.

[30] Heerman WJ, Lounds-Taylor J, Mitchell S, Barkin SL (2018). Validity of the toddler feeding questionnaire for measuring parent authoritative and indulgent feeding practices which are associated with stress and health literacy among Latino parents of preschool children. Nutrition research (New York, NY).

[31] Heller RL, Chiero JD, Trout N, Mobley AR (2021). A qualitative study of providers' perceptions of parental feeding practices of infants and toddlers to prevent childhood obesity. BMC Public Health.

[32] Vinke PC, Tigelaar C, Küpers LK, Corpeleijn E (2021). The Role of Children's Dietary Pattern and Physical Activity in the Association Between Breastfeeding and BMI at Age 5: The GECKO Drenthe Cohort. Matern Child Health J.

[33] Charney E, Goodman HC, McBride M, Lyon B, Pratt R. Childhood antecedents of adult obesity. Do chubby infants become obese adults? The New England journal of medicine. 1976; 295(1):6–9. 10.1056/nejm197607012950102 10.1056/NEJM197607012950102 1272299

[34] Kirolos A, Goyheneix M, Kalmus Eliasz M, Chisala M, Lissauer S, Gladstone M (2022). Neurodevelopmental, cognitive, behavioural and mental health impairments following childhood malnutrition: a systematic review. BMJ Glob Health.

[35] Stabouli S, Erdine S, Suurorg L, Jankauskienė A, Lurbe E (2021). Obesity and Eating Disorders in Children and Adolescents: The Bidirectional Link. Nutrients.

[36] Racicka E, Bryńska A. Eating Disorders in children and adolescents with Type 1 and Type 2 Diabetes: prevalence, risk factors, warning signs. Psychiatria polska. 2015; 49(5):1017–24. http://doi.org/10.12740/pp/39536 10.12740/PP/39536 26688851

[37] Mahmood L, Flores-Barrantes P, Moreno LA, Manios Y, Gonzalez-Gil EM (2021). The Influence of Parental Dietary Behaviors and Practices on Children's Eating Habits. Nutrients.

[38] Faul F, Erdfelder E, Buchner A, Lang AG. Statistical power analyses using G*Power 3.1: tests for correlation and regression analyses. Behavior research methods. 2009; 41(4):1149–60. 10.3758/brm.41.4.1149 10.3758/BRM.41.4.1149 19897823

[39] Xu N, Zhang G, Xie G, Chen L, Han A, Su P. Dietary literacy questionaire for school-age children's daily diet providers and its reliability and validity. Journal of hygiene research. 2020; 49(4):546–53. 10.19813/j.cnki.weishengyanjiu.2020.04.004 10.19813/j.cnki.weishengyanjiu.2020.04.004 32928353

[40] de Onis M, Onyango AW, Borghi E, Siyam A, Nishida C, Siekmann J (2007). Development of a WHO growth reference for school-aged children and adolescents. Bull World Health Organ.

[41] Fang HY, Zhai Y, Zhao LY, Yu DM, Zhang Q, Ju LH (2018). Epidemiological characteristics of overweight and obesity in Chinese children and adolescents aged 6–17 years. Zhonghua liuxingbingxue zazhi.

[42] Tao RW, Wan YH, Zhang H, Wang YF, Wang B, Xu L (2016). Relationship between hypertension and percentage of body fat, in children of Anhui province. Zhonghua liuxingbingxue zazhi.

[43] Kumar A, Sharma E, Marley A, Samaan MA, Brookes MJ (2022). Iron deficiency anaemia: pathophysiology, assessment, practical management. BMJ Open Gastroenterol.

[44] Liu J, Yuan E, Zhang Z, Jia L, Yin Z, Meng X (2012). Age- and sex-specific reference intervals for blood copper, zinc, calcium, magnesium, iron, lead, and cadmium in infants and children. Clin Biochem.

[45] Begley A, Butcher LM, Bobongie V, Dhaliwal SS (2019). Identifying Participants Who Would Benefit the Most from an Adult Food-literacy Program. Int J Environ Res Public Health.

[46] Lee HA, Park H (2017). The mediation effect of individual eating behaviours on the relationship between socioeconomic status and dietary quality in children: the Korean National Health and Nutrition Examination Survey. Eur J Nutr.

[47] Zanobini P, Lorini C, Lastrucci V, Minardi V, Possenti V, Masocco M (2021). Health Literacy, Socio-Economic Determinants, and Healthy Behaviours: Results from a Large Representative Sample of Tuscany Region, Italy. Int J Environ Res Public Health.

[48] de Jong E, Visscher TL, HiraSing RA, Seidell JC, Renders CM (2015). Home environmental determinants of children's fruit and vegetable consumption across different SES backgrounds. Pediatr Obes.

[49] Pearson N, Biddle SJ, Gorely T (2009). Family correlates of fruit and vegetable consumption in children and adolescents: a systematic review. Public Health Nutr.

[50] Chen S, Binns CW, Maycock B, Liu Y, Zhang Y (2014). Prevalence of dietary supplement use in healthy pre-school Chinese children in Australia and China. Nutrients.

[51] Kang M, Kim DW, Jung HJ, Shim JE, Song Y, Kim K (2016). Dietary Supplement Use and Nutrient Intake among Children in South Korea. J Acad Nutr Diet.

[52] Melse-Boonstra A (2020). Bioavailability of Micronutrients From Nutrient-Dense Whole Foods: Zooming in on Dairy, Vegetables, and Fruits. Front Nutr.

[53] Sanders LM, Perrin EM, Yin HS, Delamater AM, Flower KB, Bian A (2021). A Health-Literacy Intervention for Early Childhood Obesity Prevention: A Cluster-Randomized Controlled Trial. Pediatrics.

[54] Herle M, Fildes A, Steinsbekk S, Rijsdijk F, Llewellyn CH (2017). Emotional over- and under-eating in early childhood are learned not inherited. Sci Rep.

[55] Herle M, Fildes A, Llewellyn CH (2018). Emotional eating is learned not inherited in children, regardless of obesity risk. Pediatr Obes.

[56] Herle M, Fildes A, Rijsdijk F, Steinsbekk S, Llewellyn C (2018). The Home Environment Shapes Emotional Eating. Child Dev.

[57] Cole NC, An R, Lee SY, Donovan SM (2017). Correlates of picky eating and food neophobia in young children: a systematic review and meta-analysis. Nutr Rev.

[58] Steinsbekk S, Bonneville-Roussy A, Fildes A, Llewellyn CH, Wichstrøm L (2017). Child and parent predictors of picky eating from preschool to school age. The international journal of behavioral nutrition and physical activity.

[59] Vandyousefi S, Gross RS, Katzow MW, Scott MA, Messito MJ (2021). Infant and Early Child Appetite Traits and Child Weight and Obesity Risk in Low-Income Hispanic Families. J Acad Nutr Diet.

[60] Meule A, Lutz AP, Vögele C, Kübler A (2014). Impulsive reactions to food-cues predict subsequent food craving. Eat Behav.

[61] Malik VS, Schulze MB, Hu FB (2006). Intake of sugar-sweetened beverages and weight gain: a systematic review. Am J Clin Nutr.

[62] Nittari G, Scuri S, Petrelli F, Pirillo I, di Luca NM, Grappasonni I. Fighting obesity in children from European World Health Organization member states. Epidemiological data, medical-social aspects, and prevention programs. La Clinica terapeutica. 2019; 170(3):e223–30. http://doi.org/10.7417/ct.2019.2137 10.7417/CT.2019.2137 31173054

[63] Glover M, Wong SF, Taylor RW, Derraik JGB, Fa'alili-Fidow J, Morton SM (2019). The Complexity of Food Provisioning Decisions by Māori Caregivers to Ensure the Happiness and Health of Their Children. Nutrients.

[64] Katz DL, Katz CS, Treu JA, Reynolds J, Njike V, Walker J (2011). Teaching healthful food choices to elementary school students and their parents: the Nutrition Detectives™ program. J Sch Health.

[65] Parekh N, Khalife G, Hellmers N, D'Eramo MG (2020). The Healthy Eating and Living Against Noncommunicable Diseases Study: An Innovative Family-Based Intervention. Diabetes Educ.

