# Supplementary material for: Association between the dietary literacy of children's daily diet providers and school-age children's nutritional status and eating behaviours: a cross-sectional study
Source: BMC Public Health. 2022 Dec 6;22:2286. doi: 10.1186/s12889-022-14621-8 (PMC9727954; doi:10.1186/s12889-022-14621-8)
Supplement: Supplementary file 1 — Additional file 1. [file 12889_2022_14621_MOESM1_ESM.docx]

**Supplement Table 1** The Questionnaire of Children's Daily Diet Providers' Dietary Literacy (QCDDPDL)

| **Serial number** | **Items** | **Very inconsistent** | **Relatively inconsistent** | **Relatively consistent** | **Very consistent** |
| --- | --- | --- | --- | --- | --- |
|  | **Attitude** |  |  |  |  |
| 1 | Care about if your children’s diet meets nutritional needs | 0 | 1 | 2 | 3 |
| 2 | Believe that children will not become obese as long as they eat less | 0 | 1 | 2 | 3 |
| 3 | Believe that obese children are not short of nutrition | 0 | 1 | 2 | 3 |
| 4 | Pay attention to buying food for children based on the nutritional value of food | 0 | 1 | 2 | 3 |
| 5 | Pay attention to choose fresh and hygienic food | 0 | 1 | 2 | 3 |
| 6 | Be careful when cooking (less oil, less salt, less sugar) | 0 | 1 | 2 | 3 |
| 7 | Pay attention to the proper cooking method to preserve the nutritional value of food when cooking | 0 | 1 | 2 | 3 |
|  | **Action** |  |  |  |  |
| 8 | Prepare breakfast for children every day | 0 | 1 | 2 | 3 |
| 9 | Ensure that children eat regularly every day | 0 | 1 | 2 | 3 |
| 10 | Strive to make children's meals delicious | 0 | 1 | 2 | 3 |
| 11 | Interested in children's diet and nutrition books, videos, etc. | 0 | 1 | 2 | 3 |
| 12 | Make sure your children drink milk every day | 0 | 1 | 2 | 3 |
| 13 | Make sure your children eat eggs every day | 0 | 1 | 2 | 3 |
| 14 | Use different cutting boards for raw food (vegetables and raw meat) and cooked food (cooked meat, cold dishes) | 0 | 1 | 2 | 3 |
|  | **Skills** |  |  |  |  |
| 15 | Can recognize whether your child is full | 0 | 1 | 2 | 3 |
| 16 | Can distinguish the quality of food | 0 | 1 | 2 | 3 |
| 17 | Can identify expired or spoiled food | 0 | 1 | 2 | 3 |
| 18 | Can understand the nutrition label on food packaging | 0 | 1 | 2 | 3 |
| 19 | Can cook delicious meals | 0 | 1 | 2 | 3 |
| 20 | Can adjust the diet when your children feel uncomfortable | 0 | 1 | 2 | 3 |
| 21 | Know the nutritional value of daily food | 0 | 1 | 2 | 3 |
| 22 | Know how to correct children's bad eating habits | 0 | 1 | 2 | 3 |
| 23 | Know how to judge whether your children have malnutrition | 0 | 1 | 2 | 3 |
|  | **Environment** |  |  |  |  |
| 24 | Can keep home dining table and tableware clean | 0 | 1 | 2 | 3 |
| 25 | Can keep dining environment bright and comfortable | 0 | 1 | 2 | 3 |
| 26 | Family can eat at least one meal together every day | 0 | 1 | 2 | 3 |
| 27 | Family is always happy when eating together | 0 | 1 | 2 | 3 |
| 28 | Always criticize and blame children when eating | 0 | 1 | 2 | 3 |
| 29 | Allow kids to eat and play or watch TV while eating | 0 | 1 | 2 | 3 |
| 30 | Allow children to leave the table casually while eating | 0 | 1 | 2 | 3 |

Items 2, 3, 28, 29, 30 take reverse scoring.
