# Supplementary material for: Association between the dietary literacy of children's daily diet providers and school-age children's nutritional status and eating behaviours: a cross-sectional study
Source: BMC Public Health. 2022 Dec 6;22:2286. doi: 10.1186/s12889-022-14621-8 (PMC9727954; doi:10.1186/s12889-022-14621-8)
Supplement: Supplementary file 2 — Additional file 2. [file 12889_2022_14621_MOESM2_ESM.docx]

**Supplement Table 2** BMI Standard for Nutritional Status of Children Aged 7-18 in China

| **Age**  **(year)** | **Boys** | | | |  | **Girls** | | | |
| --- | --- | --- | --- | --- | --- | --- | --- | --- | --- |
|  | **wasting** | **normal** | **overweight** | **obesity** |  | **wasting** | **normal** | **overweight** | **obesity** |
| **7 ~** | ≤ 13.9 | 14.0~17.3 | 17.4~19.1 | ≥ 19.2 |  | ≤ 13.4 | 13.5~17.1 | 17.2~18.8 | ≥ 18.9 |
| **8 ~** | ≤ 14.0 | 14.1~18.0 | 18.1~20.2 | ≥ 20.3 |  | ≤ 13.6 | 13.7~18.0 | 18.1~19.8 | ≥ 19.9 |
| **9 ~** | ≤ 14.1 | 14.2~18.8 | 18.9~21.3 | ≥ 21.4 |  | ≤ 13.8 | 13.9~18.9 | 19.0~20.9 | ≥ 21.0 |
| **10 ~** | ≤ 14.4 | 14.5~19.5 | 19.6~22.4 | ≥ 22.5 |  | ≤ 14.0 | 14.1~19.9 | 20.0~22.0 | ≥ 22.1 |
| **11 ~** | ≤ 14.9 | 15.0~20.2 | 20.3~23.5 | ≥ 23.6 |  | ≤ 14.3 | 14.4~21.0 | 21.1~23.2 | ≥ 23.3 |
| **12 ~** | ≤ 15.4 | 15.5~20.9 | 21.0~24.6 | ≥ 24.7 |  | ≤ 14.7 | 14.8~21.8 | 21.9~24.4 | ≥ 24.5 |
| **13 ~** | ≤ 15.9 | 16.0~21.8 | 21.9~25.6 | ≥ 25.7 |  | ≤ 15.3 | 15.4~22.5 | 22.6~25.5 | ≥ 25.6 |
| **14 ~** | ≤ 16.4 | 16.5~22.5 | 22.6~26.3 | ≥ 26.4 |  | ≤ 16.0 | 16.1~22.9 | 23.0~26.2 | ≥ 26.3 |
| **15 ~** | ≤ 16.9 | 17.0~23.0 | 23.1~26.8 | ≥ 26.9 |  | ≤ 16.6 | 16.7~23.3 | 23.4~26.8 | ≥ 26.9 |
| **16 ~** | ≤ 17.3 | 17.4~23.4 | 23.5~27.3 | ≥ 27.4 |  | ≤ 17.0 | 17.1~23.6 | 23.7~27.3 | ≥ 27.4 |
| **17 ~** | ≤ 17.7 | 17.8~23.7 | 23.8~27.7 | ≥ 27.8 |  | ≤ 17.2 | 17.3~23.7 | 23.8~27.6 | ≥ 27.7 |
